# Role of nuclear factor-kappa B in feline injection site sarcoma

**DOI:** 10.1186/s12917-019-2100-9

**Published:** 2019-10-25

**Authors:** Cheng-Shun Hsueh, Ching-Ho Wu, Cheng-Hsin Shih, Jason Lih-Seng Yeh, Chian-Ren Jeng, Victor Fei Pang, Hue-Ying Chiou, Hui-Wen Chang

**Affiliations:** 1 Graduate Institute of Molecular and Comparative Pathobiology, School of Veterinary Medicine, National Taiwan University, No. 1, Sec. 4, Roosevelt Rd, Taipei, 10617 Taiwan; 2 Institute of Veterinary Clinical Science, School of Veterinary Medicine, National Taiwan University, Taipei, 10617 Taiwan; 3 School of Veterinary Medicine, National Taiwan University, Taipei, 10617 Taiwan; 4 Graduate Institute of Veterinary Pathobiology, College of Veterinary Medicine, National Chung Hsing University, Taichung, 402 Taiwan

**Keywords:** Feline injection site sarcoma, NF-κB inhibitor, Chronic inflammation

## Abstract

**Background:**

Chronic inflammation has been implicated in sarcomagenesis. Among various factors, activation of nuclear factor-kappa B (NF-κB) signaling pathway has been documented being able to target genes associated with tumor progression and up-regulate the expression of tumor-promoting cytokines and survival genes in several human solid tumors. Feline injection sites sarcomas (FISS) are malignant entities derived from the mesenchymal origin. The disease has been considered to be associated with vaccine adjuvant, aluminum, which serves as a stimulus continuously inducing overzealous inflammatory and immunologic reactions. To understand the contribution of NF-κB in FISS, detection of activated NF-κB in paraffin-embedded specimens, in vitro establishment of primary cells derived from FISS, and evaluation of the effects of the NF-κB inhibitor, dehydroxymethylepoxyquinomicin (DHMEQ), on primary tumor cells were conducted.

**Results:**

In this study, nuclear expression of NF-κB p65 was detected in 83.3% of FISS cases and not correlated with tumor grading, sex, and age. Primary cells derived from FISS in three cats exhibiting same immunohistochemical characteristics as their original tumor were successfully established. The NF-κB inhibitor, DHMEQ, was able to prevent nuclear translocation of NF-κB p65, inhibit cell proliferation, migration, and colonization in dosage-dependent manners, and induce cell apoptosis in these primary FISS cells.

**Conclusions:**

High expression rate of nuclear NF-κB p65 in FISS cases and dose-dependent inhibitory effects on the growth of FISS primary cells treated with NF-κB inhibitor suggested that NF-κB might be a potential molecular therapeutic target for FISS.

## Background

Feline injection site sarcomas (FISSs) were first identified in 1992 [1], as evidenced by the increase in the disease incidence after the introduction of laws requiring rabies vaccination of cats. Nowadays, FISSs is one of the most commonly diagnosed cutaneous neoplasms in cats, with a prevalence ranging from five cases per 10,000 to one case per 1000 [2–4]. The most common histologic subtype of FISS is highly invasive fibrosarcomas, but other subtypes such as rhabdomyosarcomas, myxosarcomas, chondrosarcomas, undifferentiated sarcomas, and malignant fibrous histiocytomas are reported [2, 5]. Histologically, most cases are associated with chronic inflammation characterized by perivascular aggregates of the lympho-plasma cells at the periphery of the tumor, and some cases present extensive fibrotic lesions intermixed with plump macrophages containing blue-gray cytoplasm, which is presumably the phagocytosed adjuvant materials [1, 6]. Because of these findings, the most widely accepted hypothesis for the pathogenesis of FISS involves chronic, long-term inflammation in response to the vaccination, especially with the aluminum adjuvant [2, 3, 5, 7, 8].

In fact, the concept that chronic inflammation links to the development of neoplasia has been proposed in 1863 by Rudolf Virchow [9]. Numerous mediators in response to chronic inflammation, such as cytokines, chemokines, growth factors and reactive oxygen and nitrogen species, could initiate DNA mutations and then provoke tumorigenesis through expression of critical signaling pathways [10, 11]. Transcription factors, including hypoxia-inducible factor 1 (HIF-1), signal transducer and activator of transcription 3 (STAT-3), and nuclear factor-kappa B (NF-κB), are involved in a malignant transforming network of great complexity [9, 12, 13].

The NF-κB protein family consists of five members, p65 (RelA), RelB, c-Rel, p105 (p50), and p100 (p52), which exist as homo- or heterodimers [11, 14]. Activation of the NF-κB signaling pathway canonically starts from stimulation by lipopolysaccharides (LPS), tumor necrosis factor α (TNFα), and interleukin-1 (IL-1), which can then activate their corresponding receptors and lead to the sequential recruitment of various adapters. A variety of adapter proteins and signaling kinases are responsible for subsequent recruitment and activation of IκB kinase (IKK) complex, which followed by phosphorylation of IκB family members serving as inhibitory molecules bound to NF-κB dimers. This phosphorylation is a prerequisite for subsequent polyubiquitination, which in turn leads to proteasomal disintegration of IκB. Activated NF-κB homo- or heterodimers can then translocate to the nucleus and activate target genes coding for TNF, IL-1, c-MYC, Cyclin D1, BCL-X_L_, VEGF, and COX-2, among others, which are associated with cell growth, survival, differentiation, inflammatory responses, metastasis, angiogenesis, and the regulation of apoptosis [11, 12, 14, 15].

Several strategies have been explored to inhibit the NF-κB signaling pathway, including antibodies targeting pro-inflammatory cytokine receptors (mainly anti-TNF), and agents against IKK, directed against proteasome components and transcription factors and blocking nuclear translocation [16]. With the broad effects of NF-κB, most NF-κB inhibitors bring side effects, such as bone marrow toxicity identified in animal studies, since hematopoietic cells rely on the NF-κB signaling pathway [17]. However, a NF-κB inhibitor, dehydroxymethylepoxyquinomicin (DHMEQ), a derivative of the fungal antibiotic compound, epoxyquinomicin C, has been shown exhibiting anti-inflammatory and anticancer effects through the specific suppression of nuclear translocation of p65(RelA) and c-Rel (mainly p65) [18, 19]. The anticancer effects of DHMEQ have been reported in various in vitro and in vivo studies in mouse and human cells, and revealed no significant toxicity in vivo in mice [20–28].

In feline sarcoma, the roles of NF-κB have not been widely investigated to date. To better understand the importance of NF-κB activation in the pathogenesis of FISS, we evaluated the overall expression rate of nuclear localized NF-κB p65 protein in formalin-fixed, paraffin-embedded (FFPE) specimens, followed by in vitro establishment of primary cells derived from FISS for efforts to realize the functional effects of NF-κB through administering the NF-κB inhibitor, DHMEQ, to these cells. In this study, we were able to show that FISS exhibited canonical activation of the NF-κB pathway due to p65 expression by both immunohistochemistry (IHC) and immunocytochemistry (ICC) staining, and we also demonstrated that a NF-κB inhibitor, DHMEQ, significantly inhibited cell proliferation, migration, and colonization, and induces apoptosis of the tumor cells. These findings, for the first time, support the activation of NF-κB in FISS, and imply potential therapeutic strategies through blocking the signaling pathway of NF-κB.

## Results

### NF-κB p65 was highly expressed in the nucleus of FISS cells in FFPE sections, and not correlated with histopathological grading and clinical variables

**Fig. 1:**
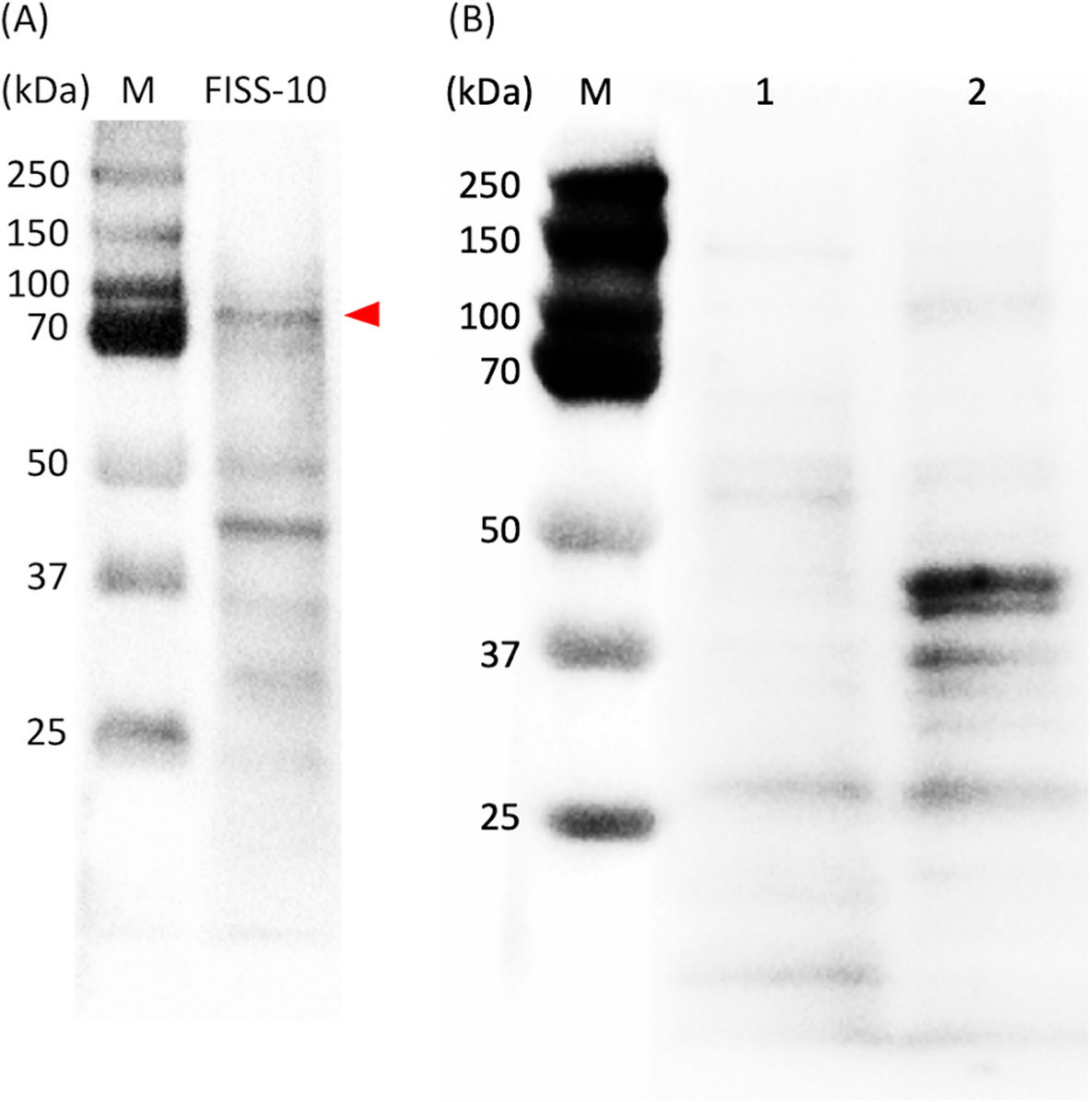
Western blot detection of the nuclear factor-kappa B using rabbit polyclonal NF-κB p65 (clone ab86299, Abcam) antibody. **a** A distinct band migrated to the size about 70 kDa (marked with arrowhead) was detected. **b** Normal feline spinal cord (1) and skeletal muscles (2) served as negative controls. No signal was observed at the size of 70 kDa

**Fig. 2:**
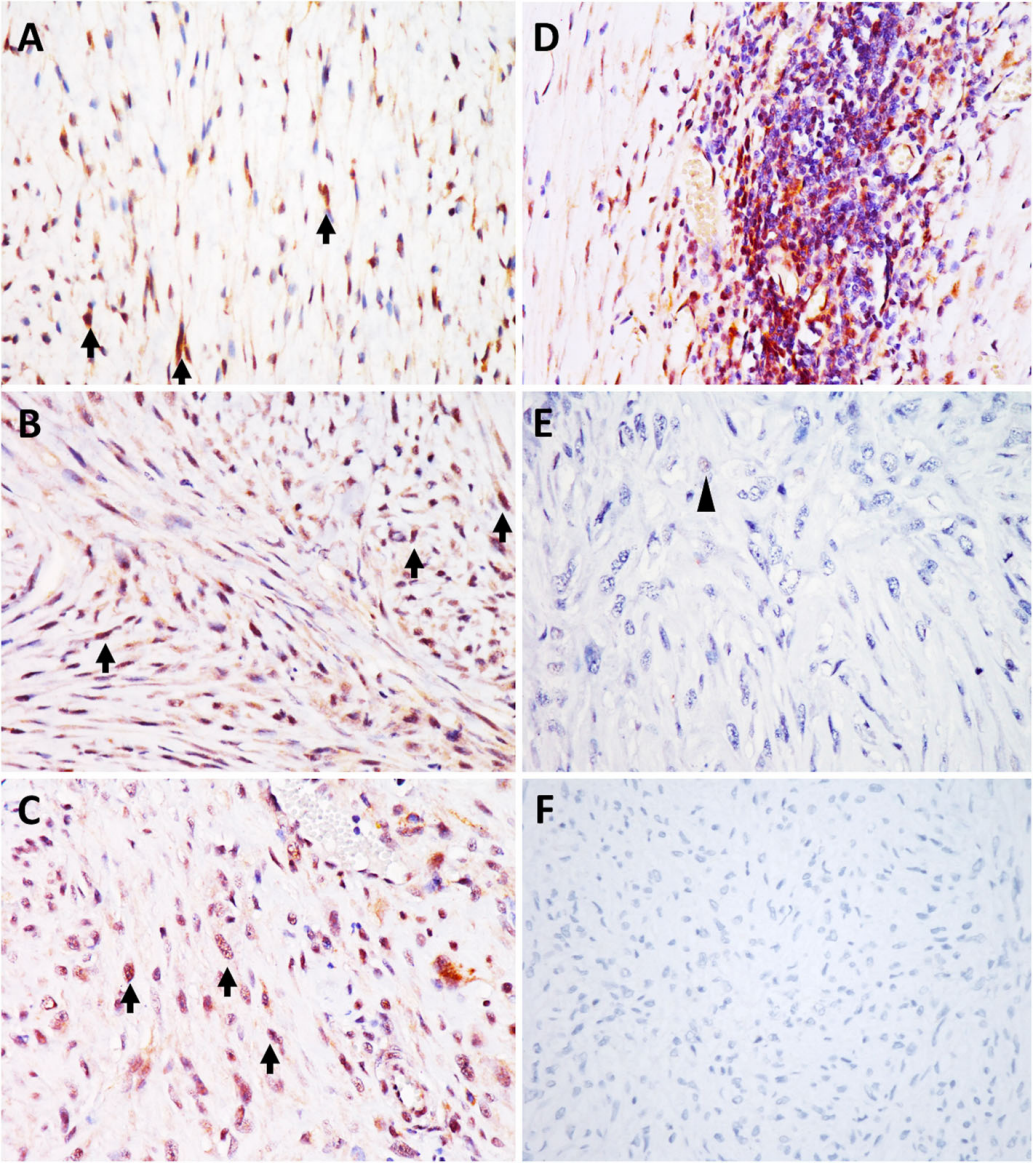
Detection of NF-κB p65 in feline injection site sarcomas (FISSs) by immunohistochemistry assay (IHC). Unequivocal brown nuclear NF-κB staining (arrows) in at least 5% of tumor cells were designated as positivity. In NF-κB p65-positive FISS cases, the expression of NF-κB p65 was consistent without distinct variation. **a** NF-κB p65-positive, grade I FISS. **b** NF-κB p65-positive, grade II FISS. **c** NF-κB p65-positive, grade III FISS. **d** Lymphoid aggregates peripheral to the neoplasm expressed nuclear NF-κB p65 subunits. **e** NF-κB p65-negative, grade III FISS. Nuclear signals (arrowhead) presented in less than 5% of neoplastic cells were designated as negativity. **f** Negative control

In this retrospective study, clinicopathological data are tabulated in Table 1. Median age was 9 ± 2.9 (range, 3–16) years and 48% were male. All tumors were localized at the injection site in a variety of locations (Table 1). Histopathologically, according to the soft tissue sarcoma grading system, 16.7% (*n* = 7), 45.2% (*n* = 19) and 38.0% (*n* = 16) of the FISSs were graded as grades I, II, and III, respectively. After confirming the specificity of the NF-κB p65 polyclonal antibody in feline cells by western blotting (Fig. 1a), IHC was performed for detecting the expression of NF-κB p65 in FISS tissues. In IHC staining, approximately 83.3% (*n* = 35) of the cases were positive (> 5% of the nuclei staining) for NF-κB p65 (Fig. 2). Lymphoid aggregates peripheral to the neoplasm also strongly expressed nuclear NF-κB p65 subunits (*n* = 36) (Fig. 2). Notably, NF-κB p65 activity in FISSs was not correlated with tumor grading, sex and age (*P* > 0.05).

**Table 1:** Clinical data and immunohistochemical result of NF-κB p65 from 42 feline injection site sarcomas

	Breed	Sex	age (year)	Location^a^	Grading	Expression of nuclear NF-κB p65^b^
1	Unknown	F	16	Dorsal neck	III	+
2	Mixed	Fsp	11	Interscapula	III	+
3	Mixed	Fsp	10	Back	II	+
4	Mixed	Mc	9	Back	II	+
5	Mixed	Fsp	11	Right thigh	III	+
6	Mixed	Mc	6	Back	III	+
7	Persian	Fsp	8	Interscapula	II	+
8	Mixed	F	3	Interscapula	II	–
9	Persian	Fsp	13	Back	III	+
10	Mixed	M	8	interscapula	II	–
11	Mixed	Fsp	10	Back	I	–
12	Mixed	Mc	10	Interscapula	II	+
13	Mixed	Fsp	10	Back	III	+
14	Mixed	M	3	Back	III	–
15	Mixed	Mc	10	Back	II	+
16	Mixed	Fsp	11	Left scapula	III	+
17	Scottish Fold	Fsp	8	Dorsal neck	II	+
18	Mixed	Mc	5	Back	I	+
19	Mixed	F	12	Back	III	+
20	Mixed	Mc	11	Back	II	+
21	Persian	Mc	7	Right scapula	III	–
22	Mixed	Mc	11	Right scapula	II	–
23	Mixed	Fsp	11	Dorsal neck	I	+
24	Mixed	Mc	7	Interscapula	III	–
25	Mixed	M	5	Back	I	+
26	Mixed	Fsp	8	Back	II	+
27	Mixed	Mc	9	Interscapula	II	+
28	Mixed	Fsp	9	Interscapula	II	+
29	Mixed	Fsp	10	Dorsal neck	I	+
30	Mixed	M	8	Back	II	+
31	Persian	F	12	Back	III	+
32	Mixed	Mc	11	Left scapula	II	+
33	Mixed	Mc	6	Back	I	+
34	Mixed	Fsp	12	Interscapula	II	+
35	Unknown	F	12	Dorsal neck	II	+
36	Mixed	Mc	Unknown	Right scapula	II	+
37	Mixed	Mc	5	Right scapula	II	+
38	Mixed	Fsp	5	Back	I	+
39	Mixed	Fsp	5	Right scapula	III	+
40	Mixed	M	5	Right scapula	III	+
41	Mixed	M	10	Dorsal neck	III	+
42	Mixed	F	10	Back	III	+

*Abbreviations*: *M* male, *Mc* male castrated, *F* female, *Fsp* female spayed

^a^Locations are based on the history in the histopathology submission form, and dorsal cervical, thoracic and lumbar regions might be referred to as back

^b^- = negative; + = more than 5% cells positive

### Immunophenotypes of FISS cells, FISS-07, FISS-08, and FISS-10, were consistent with corresponding FFPE specimens; and NF-κB inhibitor DHMEQ inhibited nuclear translocation of p65 NF-κB

**Fig. 3:**
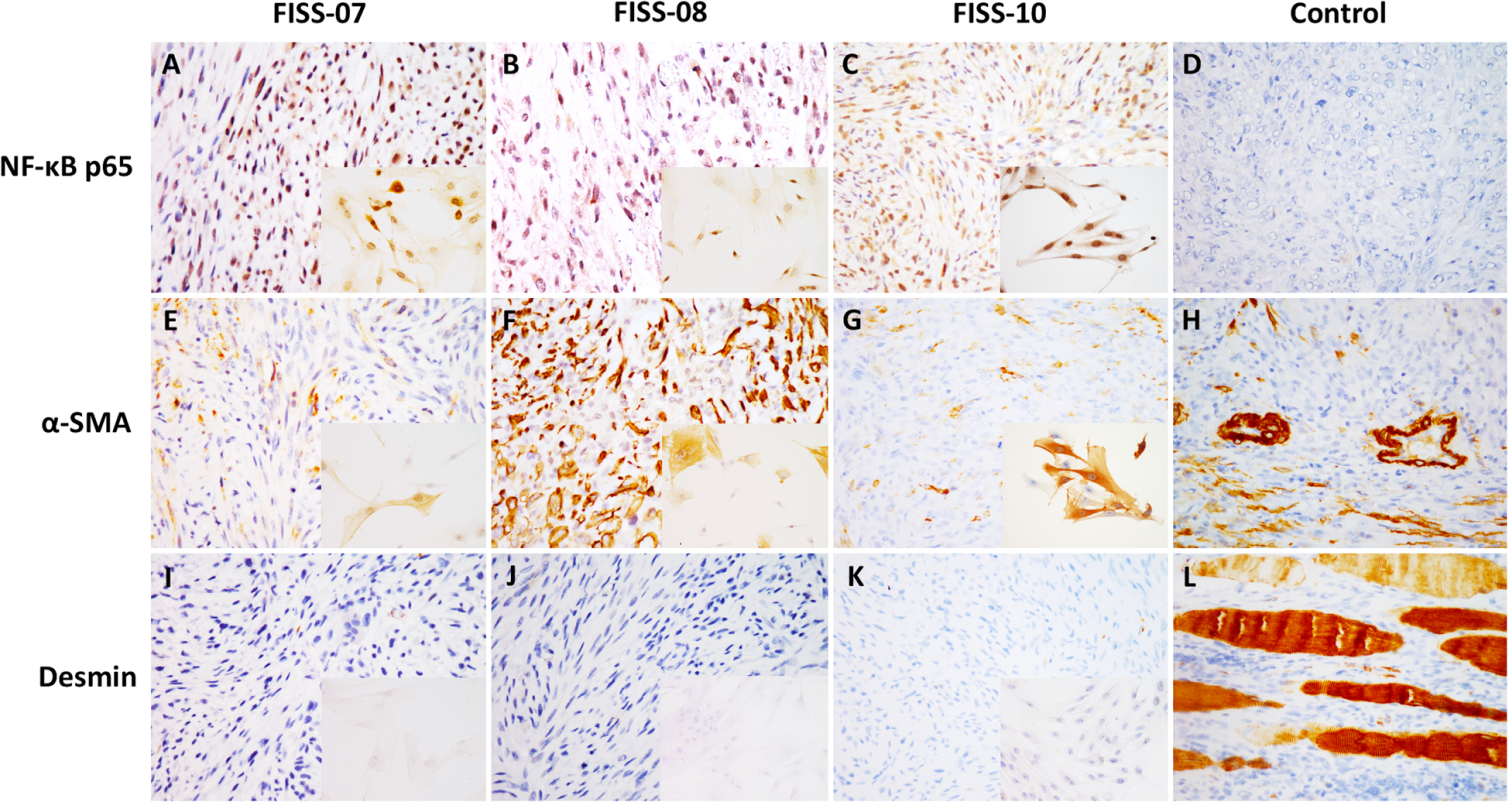
Correlation of immune phenotypes in FFPE sections and cell cultures of FISSs. Neoplastic cells of FISS-07 (**a**), −08 (**b**), and − 10 (**c**) in both FFPE and cell cultures (Inset) displayed nuclear signals for NF-κB p65. Neoplastic cells of FISS-07 (**e**), − 08 (**f**), and − 10 (**g**) in both FFPE and cell cultures (Inset) showed heterogeneous positive signals for α-SMA. Neoplastic cells of FISS-07 (**i**), − 08 (**j**), and − 10 (**k**) are negative for desmin. Inset, cell cultures. **d** Negative control. **h** Normal blood vessels were used as a positive control tissue for α-SMA expression. **l** Normal skeletal muscles were used as a positive control tissue for desmin expression

**Fig. 4:**
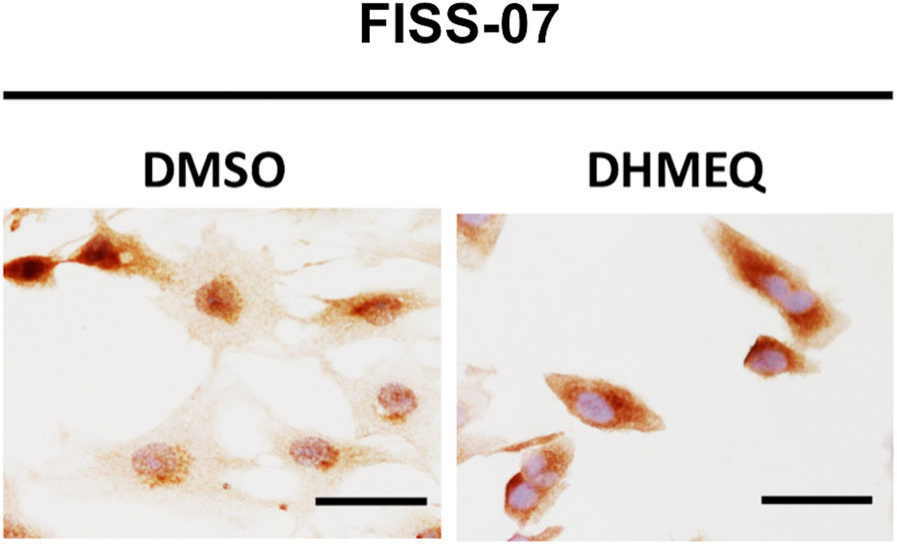
Effects of DHMEQ on nuclear translocation of NF-κB p65 in FISS-07. Cells were treated with or without 10 μg/ml of DHMEQ for 3 h and subjected to ICC with anti-NF-κB p65 antibody. The results indicated that DHMEQ significantly inhibited nuclear translocation of NF-κB (brown, NF-κB p65; blue, nuclei). Scale bars = 50 μm

Three FISS cells, FISS-07, FISS-08, and FISS-10, derived from cat 40, 41, and 42 were established, respectively. Both ICC and IHC stainings using the same antibodies were intended for characterization and identification of the cell cultures and FFPE samples from these three cats. The results are shown in Table 2 and Fig. 3. Overall, these three cases (FISS-07, FISS-08, and FISS-10) had the similar ICC/IHC profile to their corresponding FFPE specimens. Interestingly, these tumor cells in ICC/IHC were all immunoreactive for α-smooth muscle actin (α-SMA), but the immune labeling was heterogeneously distributed throughout the FFPE samples, as well as the cell cultures. Neoplastic cells in FFPE samples and cell cultures in these three cases were negative for desmin. Positivity of α-SMA and negativity of desmin, taken together, are able to conclude the diagnosis of these three cases as myofibroblast-rich sarcoma. Diffuse strong nuclear and cytoplasmic signals of the p65 NF-κB subunit were detected in neoplastic cells in both FFPE samples and cell cultures, indicating activation of the p65 NF-κB subunit in these three cases. After application of NF-κB inhibitor DHMEQ to tumor cells, as expected, nuclear translocation of p65 NF-κB was successfully suppressed (Fig. 4). At a concentration of 10 μg/ml, strong positive signals could be exclusively detected in the cytoplasm in FISS-07, FISS-08 and FISS-10.

**Table 2:** Clinical data, pathological features and immunologic profile in 3 FISSs with in vitro establishment of primary cells

	FISS-07	FISS-08	FISS-10
Breed	Mixed	Mixed	Mixed
Sex/Age (years)	Male/5y	Male/10y	Female/10y
Tumor size	< 5 cm	> 5 cm	< 5 cm
Location	Right scapula	Dorsal neck	Back
Pathological diagnosis	Sarcoma	Sarcoma	Sarcoma
Grading	III	III	III
IHC/ICC profile^a^			
α-SMA	± /±	±/ ±	±/±
Desmin	−/−	−/−	−/−
NF-κB	+/+	+/+	+/+

*Abbreviations*: *IHC* immunohistochemistry, *ICC* immunocytochemistry, *α-SMA* alpha-smooth muscle actin, *NF-κB* nuclear factor-kappa B

^a^-: negative; ±: present as heterogeneous pattern; +: more than 5% cells positive

### DHMEQ inhibited cell proliferation and induced cell death in FISS cells in a dose-dependent manner

**Fig. 5:**
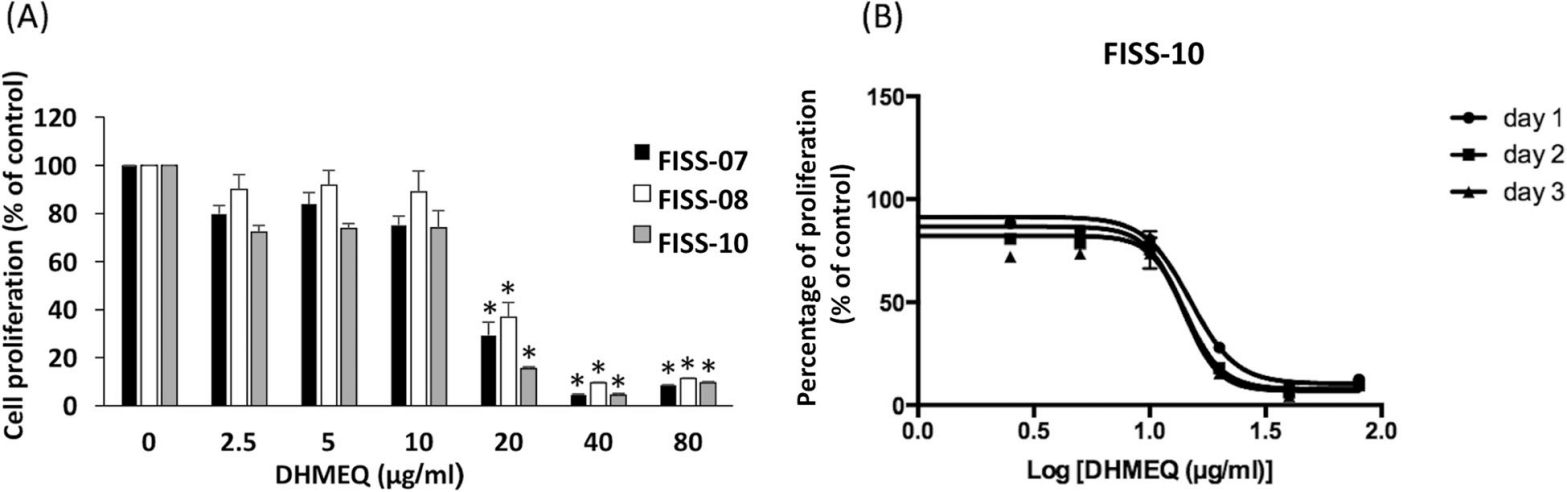
DHMEQ significantly inhibited cell proliferation in FISS cells, as evaluated by Alamar Blue assays. **a** FISS-07, FISS-08 and FISS-10 were treated with serial doses of DHMEQ for 72 h in triplicate. Significant inhibitory effects presented at doses of 20 μg/ml and above. Data are presented as means ± SD. *, statistically significant than controls (*P* < 0.05). **b** FISS-10 cells were incubated with varying concentrations of DHMEQ for 24, 48 and 72 h. Alamar Blue assays demonstrated dose-dependent growth inhibition of FISS cells in response to DHMEQ treatment

**Fig. 6:**
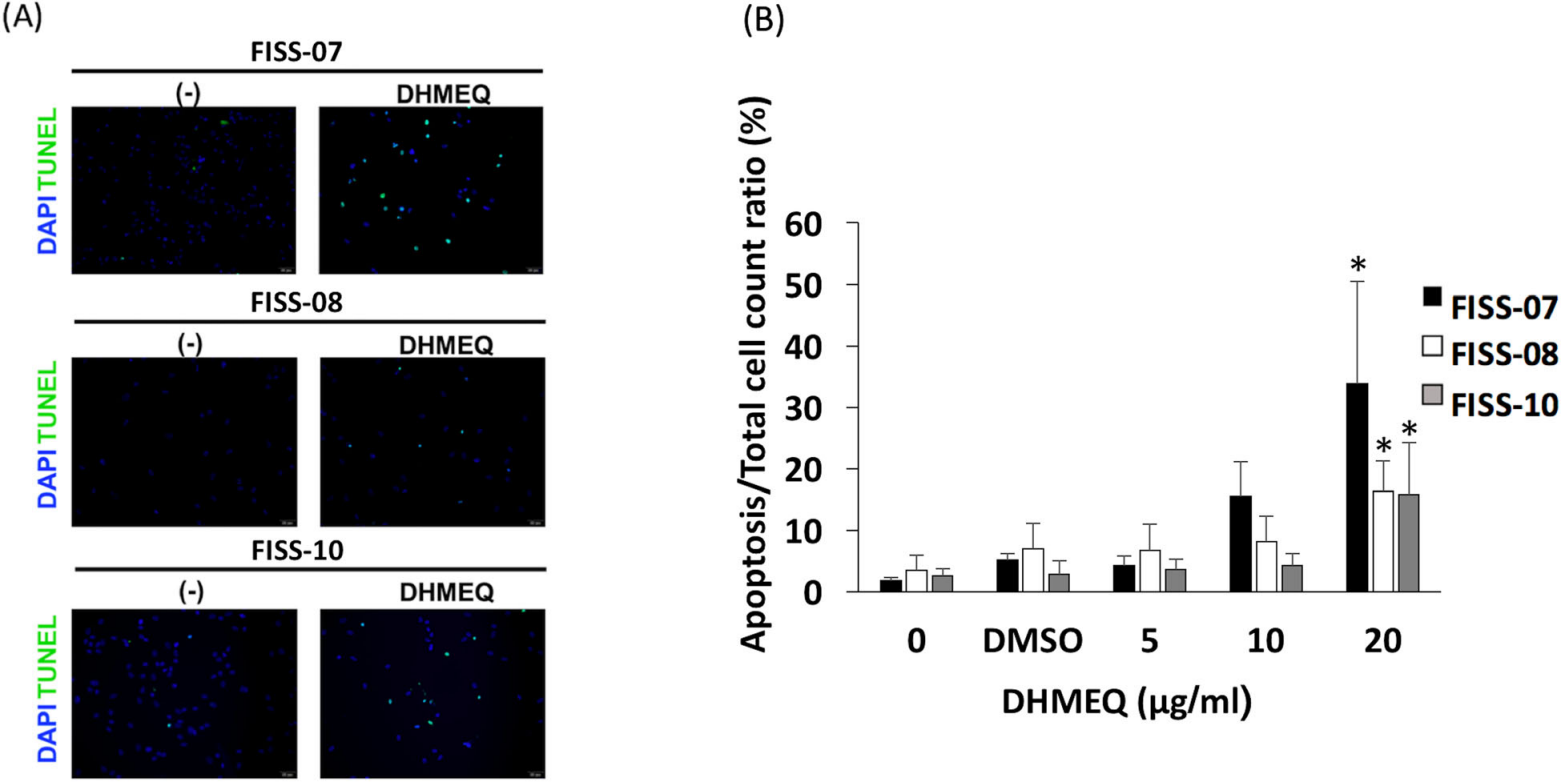
FISS-07, FISS-08 and FISS-10 cells were incubated with or without DHMEQ for 24 h, and the cells underwent apoptosis, as detected by TUNEL assays. **a** The panels show merged images of nuclei stained with DAPI (shown in blue) and 3′-OH DNA ends stained with fluorescein (in green) in FISS cells treated with 20 μg/ml DHMEQ. **b** DHMEQ induced apoptosis in these FISS cells in a dose-dependent manner. FISS-07 showed a greater extent of apoptosis compared to its control groups and FISS-08 and FISS-10. *, statistically significant than controls (*P* < 0.05)

The DHMEQ was able to inhibit cell proliferation in a dosage-dependent manner in FISS cells (Fig. 5a) (*P* < 0.05). When cells were treated with DHMEQ, significant growth inhibition was observed at concentrations of 20, 40 and 80 μg/ml. The inhibitory effect at 72 h was slightly greater than those at 48 h and 24 h. The inhibitory effects of DHMEQ were evaluated by nonlinear regression analysis, which all fitted to a sigmoidal dose–response curve (*R*^*2*^ > 0.98) (Fig. 5b). Overall, FISS-10 at 72 h showed the highest sensitivity (IC50 value, 14.15 ± 2.87 μg/ml), followed by FISS-07 (IC50 value, 16.03 ± 1.68 μg/ml), and FISS-08 (IC50 value, 17.12 ± 1.19 μg/ml). All FISS cells are more sensitive to DHMEQ treatment than primary cells derived from normal feline soft tissue (IC50 value, 27.34 ± 2.87 μg/ml) (Table 3). We then examined the effects of DHMEQ on the induction of apoptosis in FISS cells. Cells grown to confluence were treated with DHMEQ for 24 h and apoptosis in these cells was detected by a terminal deoxynucleotidyl transferase dUTP nick end labeling (TUNEL)-based method. As shown in Fig. 6, DHMEQ induced cell apoptosis in these FISS cells in a dosage-dependent pattern, and all FISS cells treated with 20 μg/ml DHMEQ exhibited a greater extent of apoptosis as compared to the control groups (*P* < 0.05).

**Table 3:** IC50 concentrations for each FISS cells when treated with DHMEQ

Cell	DHMEQ 24 h (μg/ml)	DHMEQ 72 h (μg/ml)
FISS-07	20.32 ± 0.84	16.03 ± 1.68
FISS-08	18.17 ± 2.96	17.12 ± 1.19
FISS-10	14.96 ± 1.46	14.15 ± 2.87
Normal feline soft tissue	–	27.34 ± 2.13

*Abbreviations*: *DHMEQ* dehydroxymethylepoxyquinomicin; ±, Standard deviation

### DHMEQ reduced cell migration of FISS cells

**Fig. 7:**
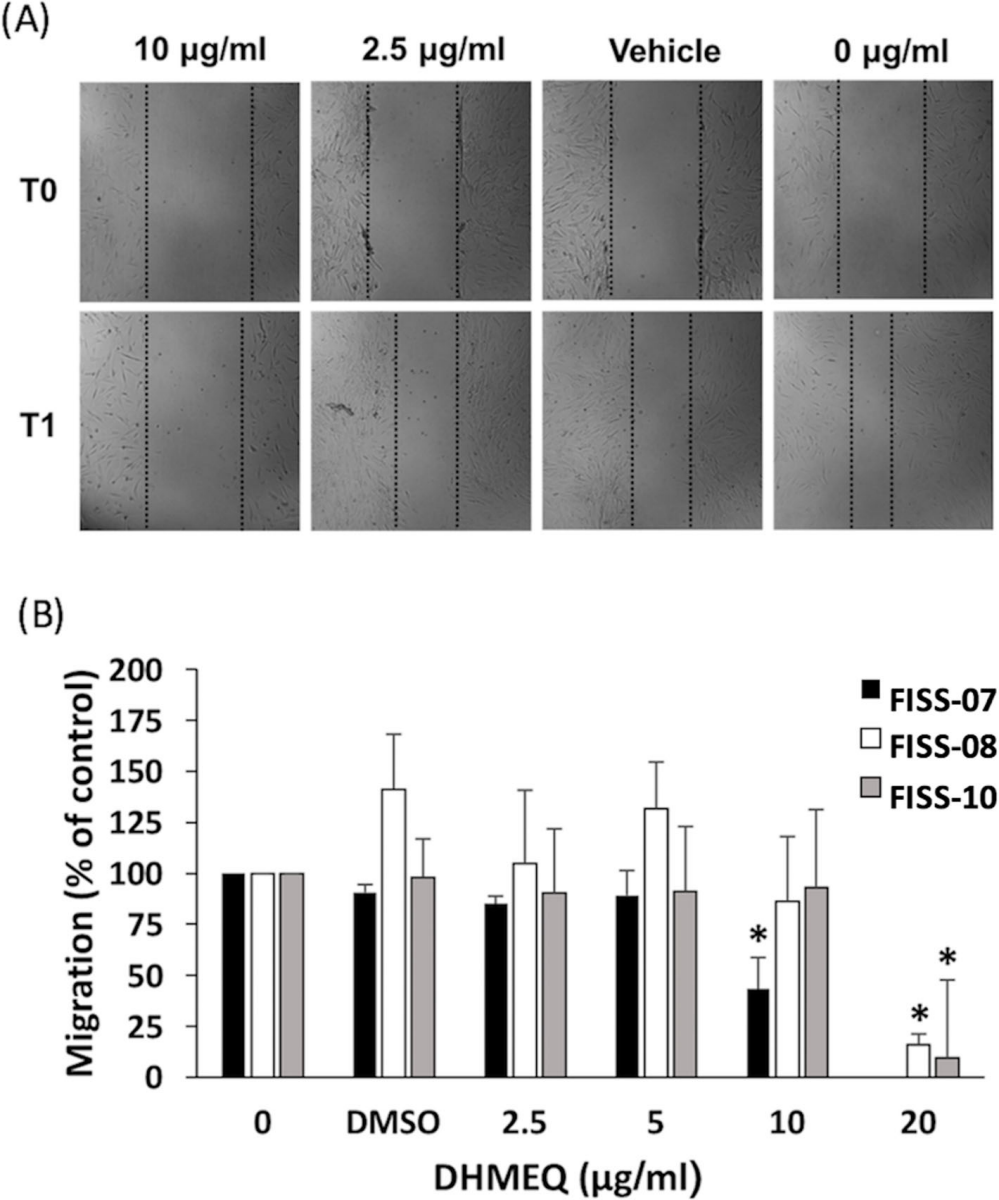
DHMEQ inhibited migration of FISS cells. Wound healing assay in FISS-07. **a** Cell starved overnight (0.1% FBS) were used to generate wounds before DHMEQ treatment for 24 h. **b** Doses of 10 and 20 μg/ml DHMEQ were able to significantly reduce cell migration of FISS-07, FISS-08, and FISS-10 cells, respectively. *, statistically significant than controls (*P* < 0.05)

To evaluate the effect of DHMEQ on cell migration, FISS cells were incubated in 0.1% serum medium overnight before generating the wound. As the FISS cells treated with the DHMEQ at concentrations higher than 20 μg/ml already resulted in cell detachment, the experiment was performed by treating FISS cells with 20, 10, 5, and 2.5 μg/ml DHMEQ, DMSO alone, and only 0.1% serum medium for 24 h. Wound healing was significantly inhibited by DHMEQ at the concentration of 10 μg/ml for FISS-07, and 20 μg/ml for FISS-08 and FISS-10 (*P* < 0.05) (Fig. 7a). The migration distance of FISS-07 cells treated with 10 μg/ml was about 50% less than that of the control groups. Similarly, the migration distance of FISS-08 and FISS-10 treated with DHMEQ at 20 μg/ml were only 16 and 10% of their control groups, respectively (Fig. 7b).

### DHMEQ inhibited colony formation of FISS cells

**Fig. 8:**
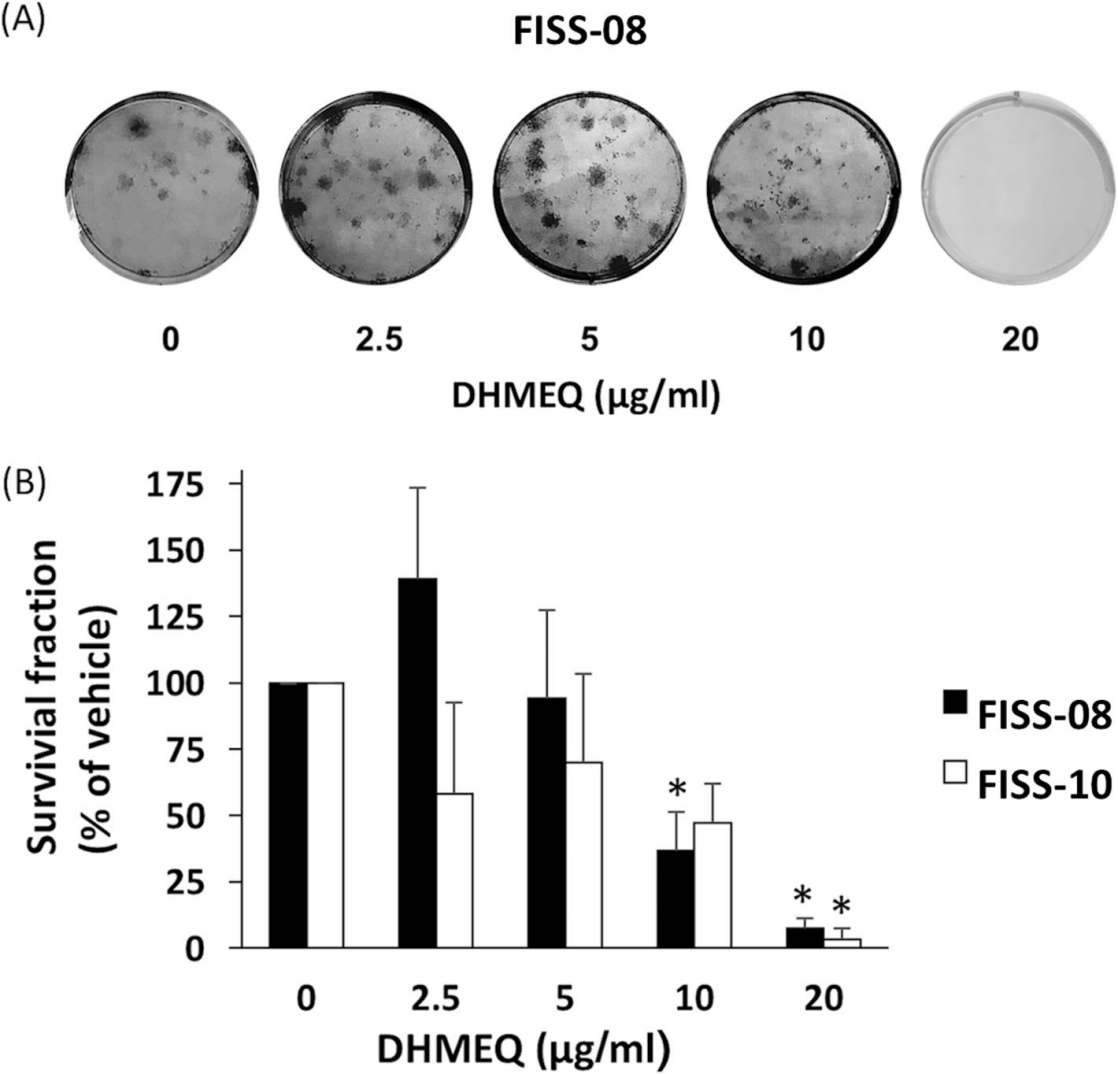
DHMEQ inhibited colony formation of FISS cells. **a** Representative photography after treatment with DHMEQ to inhibit FISS-08 colony formation. **b** FISS-08 and FISS-10 cells were treated with various concentrations of DHMEQ for 72 h. The inhibitory effect of DHMEQ at a concentration of 20 μg/ml on FISS-08, and of 10 μg/ml on FISS-10. *, statistically significant than controls (*P* < 0.05)

To evaluate the ability of DHMEQ to reduce clonogenic survival of FISS cells, a clonogenic assay was performed. FISS cells were treated with various concentrations of DHMEQ for 72 h followed by incubation with medium without drug for 3 weeks. Colonies with a minimum of 30 cells were counted. While the FISS-07 cells had a low plating efficiency (less than 0.001%) were not able to form colonies, the FISS-08 and FISS-10 cells had plating efficiency approximately 1% were successfully used to conduct the assay. Our result demonstrated that the colony formation was significantly inhibited by DHMEQ at a concentration of 20 μg/ml for FISS-08 and 10 μg/ml for FISS-10 (*P* < 0.05) (Fig. 8).

## Discussion

Activation of NF-κB has been implicated in sarcomagenesis in chronic inflammation in humans [9]. In the present study, we have demonstrated that the nuclear localized NF-κB was detected in 83.3% of FISS cases, suggesting that NF-κB might play a role in development of FISS. To establish a platform for preclinically evaluating the efficacy of anti-tumor drugs, and for studying the activated status of NF-κB in FISS, primary cells derived from FISS in three cats exhibiting similar immunohistochemical characteristics as their original tumor sites have also been established. Using this in vitro model, we have further demonstrated that the NF-κB inhibitor, DHMEQ, exhibited dose-dependent inhibitory effects on the growth of FISS primary cells and serve as a new molecular therapeutic target for FISS.

FISS is an invasive malignancy that leads to extremely high recurrence rates, ranging between 14 and 40%, even when pathologists agree the excisional margins are negative [29, 30]. Current optimal treatment of FISS consists of aggressive, wide excision with at least 3 or 5 cm margins peripherally, and two fascial plane deep in the tumor, with adjunctive therapies of radiation and chemotherapy [30–33]. Doxorubicin is the only cytotoxic drug that can prolong remission and better the quality of life of cats, to date. However, such agents do not prolong the disease-free interval [34]. Recently, with extensive and pathogenesis-based in vitro and in vivo studies, several novel small molecules, including tyrosine kinase inhibitors (TKI) [35], liposomal doxorubicin (Caelyx) [36] and glutathione-stabilized gold nanoparticles non-covalently modified with doxorubicin (Au-GSH-Dox) [37], have been proposed to be ideal for therapy. Among these small molecules, TKI (toceranib) brought no clinical response in cats with FISS [35]. Other treatments that can be commercially obtained include feline interleukin-2, expressed by a recombinant canarypox virus, which functions as an immune stimulator. The effectiveness and the prognosis is generally better than regular surgical excision [38]. Even though comprehensive strategies for improvements in therapies have been widely investigated, FISS remains a therapeutic challenge. In this retrospective study, for the first time, we were able to identify the activated form of NF-κB p65 protein in FISS tumor cells, and, interestingly, in lympho-plasmacytic cells in the tumor bed as well. The presence of lymphoid follicles has been recognized as an immune response to stimulation by vaccine adjuvants; however, it could also be a host response to FISS, in particular, the existence of peritumoral regulatory T-cells, which function to inhibit antitumor immunity through suppression of anti-tumor cytotoxic T-cells [5, 39]. NF-κB activation in these lymphoid aggregates could imply certain functions associated with tumorigenesis, since activation of NF-κB in tumor-associated inflammatory cells is evident in response to inflammatory cytokines and signals released by necrotic tumor cells that lead to induction of growth, survival and angiogenetic factors. These factors are also capable of initiating the activation of NF-κB in tumor cells, as well as establishing the proper microenvironment for tumor growth [40]. However, the exact function of lymphoid aggregates remains unknown in FISS.

In the in vitro functional assays, one of the highlights of our study is the usage of primary neoplastic cells as the model. Cell lines for drug development and basic biology research have many advantages, including reproducibility, ease of use and cost effectiveness, but may manifest genetic and phenotypic drift due to their adaptation to cell culture [41, 42]. Therefore, there is increasing realization of shortcomings in preclinical studies based on cell lines. In our study, primary cells within the first three subcultures were employed, which should closely resemble the clinical scenario with minimal genetic alteration, the results might be considerably relevant to those of in vivo models.

Under ICC staining, phosphorylated NF-κB p65 subunit usually revealed expression in both cytoplasm and nuclei of the neoplastic cells, but only nuclear signal is suggest to represent the activation of NF-κB because nuclear expression implicates post-translational modification, which is an essential step for cytoplasmic-to-nuclear localization and initiation of transcription [43]. The NF-κB inhibitor, DHMEQ as expected, was able to effectively inhibit constitutive nuclear translocation of NF-κB p65, and therefore would be expected to block downstream transcription and associated functions. In cell proliferation assays, DHMEQ caused significant inhibition of cell growth, with an IC50 of approximately 14–17 μg/ml, indicating that activation of NF-κB acts in a key role in the proliferation of these cells. Growth inhibitory effects of DHMEQ on various kinds of human tumors have been reported, including prostatic carcinoma [44], thyroid carcinoma [24], breast cancer [45], pancreatic carcinoma [46], urothelial cell carcinoma [21], nasopharyngeal carcinoma [47], adult T-cell leukemia [48], multiple myeloma [20], B-cell lymphoma [49] and others. In those reports, dose-response inhibitory effects were obtained with similar concentrations of DHMEQ to these in FISS in our study, raising a convincing and pleasing efficacy of DHMEQ in FISS cells. In order to investigate the specificity of anti-proliferative effects of DHMEQ, primary healthy cells isolated from subcutaneous soft tissues and skeletal muscles remote from the tumor site were used. The IC50 of these primary cells were higher than those of tumor cells, indicative of the inhibitory effects of DHMEQ specifically to tumor cells, despite the fact that tumor cells were obtained from high grade sarcomas, presenting a more aggressive phenotype and potential resistance to therapy.

NF-κB is known to inhibit apoptosis through the induction of anti-apoptotic proteins, and through suppression of pro-apoptotic genes [9, 15]. DHMEQ in the present study caused a dose-dependent increase in the levels of DNA breakage in FISS cells, which is a hallmark of apoptotic cell death. Furthermore, FISS-07 cells may be more dependent on the survival benefits resulting from NF-κB activation than other cells, since DHMEQ at 10 and 20 μg/ml caused a higher degree of apoptosis than in FISS-08 and FISS-10 cells. Reports have shown that DHMEQ reduced the expression levels of BCL family genes, which are responsible for suppression of apoptosis; however, the effects of DHMEQ on anti-apoptotic molecules may differ among tumors [20, 23]. In other functional assays, DHMEQ at concentrations of 10 and 20 μg/ml was able to significantly decrease cell migration in FISS-07, FISS-08 and FISS-10 and colonization in FISS-08 and FISS-10, which are key steps in tumor expansion and invasiveness. In this study, low plating efficiency of FISS-07 in the clonogenic assay is assumed to be associated with the nature biological characteristic of FISS-07. Reports have indicated, in primary cell cultures, the plating efficiency could be as few as 0.5% or even 0% [50]. Because FISS typically have a propensity toward invasiveness, on the basis of our results, we believe NF-κB inhibitor to be an efficient anticancer agent against aggressive FISS.

## Conclusion

Our study showed that activation of NF-κB is crucial for FISS, as evidenced by strong expression detected by ICC/IHC and the inhibitory and anti-proliferative effects by the NF-κB inhibitor. In anti-tumor assays, NF-κB inhibitor in our study was able to inhibit cell proliferation, migration, and colonization, and was able to induce cell apoptosis in FISS cells. DHMEQ could be a potential therapeutic compound that specifically targets activated NF-κB in FISS cells. Additionally, we believe that the established in vitro model of FISS would be useful to obtain basic evidence for testing anti-tumor properties of novel agents, which then can be sanguinely translated into preclinical medicine. To fully understand the role of NF-κB in FISS, further studies to explore the association among immuno-expression of NF-κB and prognostic variables, such as disease-free survival and overall survival time are important tasks.

## Methods

### Case collection

A total 42 archival cases from cats diagnosed with FISS or vaccine-associated sarcoma during 2014–2017 from the database of the Graduate Institute of Molecular and Comparative Pathobiology, National Taiwan University was selected for investigating expression of the activated form of NF-κB in FISS. Data tabulated from records were signalment and grading of sarcomas (Table 1). Histopathology slides and formalin-fixed, paraffin-embedded (FFPE) tissue sections were retrieved in this retrospective study.

### Cell culture

All procedures involving animal sample collection were performed in accordance with guidelines of Institutional Animal Care and Use Committee (IACUC) of National Taiwan University (NTU; Taiwan, Republic of China) and carried out under the regulation and permission of the IACUC protocol (NTU107-EL-00165) at NTU. Primary neoplastic cells derived from cases 40, 41, and 42 (Table 1) were designated as FISS-07, FISS-08, and FISS-10, respectively. These primary cells were derived from masses at the vaccination site of three cats with documented in the medical record of vaccination (Table 2). For each case, a proportion of the tumor mass was submitted for histopathological examination, and an additional portion was saved for primary cell establishment. Histologically, these cases were confirmed as feline injection site sarcomas. To establish the primary neoplastic cell cultures, the tumor mass was washed with PBS (Gibco, Grand Island, NY, USA) containing antibiotics (100 μl/ml penicillin, 100 μl/ml streptomycin, Gibco), cut into numerous small fragments (0.5 × 0.5 × 0.5 cm), and plated into a 75 T sterile tissue culture flask (CORNING, Corning, NY, USA) containing 15 ml of Dulbecco’s modified Eagle’s medium (DMEM, Gibco), supplemented with 20% fetal bovine serum (FBS, Gibco) and antibiotics. As to the preparation of primary normal feline cells, briefly, small pieces of tissues, including subcutaneous soft tissues and skeletal muscles remote from the tumor site were aseptically and gently minced into small pieces and washed with PBS containing antibiotics twice. The tissues were then digested by 0.05% trypsin (Gibco) for 10 min. The suspended pellet mixture was filtered through the pre-wet 100 μm strainer and the cells in the flow-through were collected, centrifuged, and re-suspended with DMEM (Gibco), 20% FBS (Gibco) and antibiotics (Gibco). The suspension was finally seeded onto a 75 T sterile tissue culture flask (CORNING). The culture flasks were placed in a 37 °C, humidified, 5% CO_2_ cell culture incubator for a total of approximately 10 days, and media were refreshed every 3 days. Under microscopic examination and immunocytochemistry staining, most of these primary normal cells exhibited spindle shape and tissue fibroblasts-like morphology with occasional desmin positivity. All the following functional tests were finished within a maximum of the first three passages of primary cells.

### Immunohistochemistry staining

Formalin-fixed paraffin-embedded tissue (FFPE) sections were immunostained with rabbit primary antibodies against NF-κB p65, alpha smooth muscle actin (α-SMA) and desmin. In the immunohistochemical (IHC) protocol, 4 μm thick FFPE slides were deparaffinized and rehydrated with xylene and ethanol. Antigen retrieval was applied by boiling the slides in the triplicates (Cell Marque, Rocklin, CA, USA) in a microwave at 95 °C for 10 min. The slides were then incubated with 10% protein-blocking normal goat and rabbit sera (Dako, Glostrup, Denmark) for 30 min to reduce non-specific binding. Antigen expression was detected by primary antibodies against rabbit polyclonal NF-κB p65 (clone ab86299, 1 in 200 dilution; Abcam, Cambridge, MA, USA), α-SMA (clone 1A4, 1 in 200 dilution; Dako) and desmin (clone D33, 1 in 200 dilution; Dako) for 1 h. Endogenous peroxidase activity was blocked with 3% hydrogen peroxide (KYB, New Taipei City, Taiwan) for 15 min. Afterward, primary antibodies were detected using a REAL Envision kit (Dako) using diaminobenzidine as a chromogenic substrate. The slides were finally counterstained with hematoxylin (Muto Pure Chemicals, Tokyo, Japan) for 15 s. Substitution of primary antibody with antibody diluent was performed as a negative control.

### Western blotting for NF-κB p65 antibody validation

To validate the specificity and cross-reactivity of the polyclonal NF-κB p65 antibody on feline specimens, immunoblot analyses for detecting the NF-κB p65 protein around the phosphorylation site of Serine 536 was performed. Briefly, primary cells from FISS-10 were collected, centrifuged at 1200 g for 10 min and washed twice with PBS. Several pieces of fresh, intact skeletal muscle and spinal cord from a necropsied cat died of the unrelated disease were collected, serving as negative controls. Total protein was prepared by incubating cells and homogenized tissues of skeletal muscle and spinal cord in RIPA lysis buffer (VWR Chemicals, Sohn, Ohio, USA) on ice and mixed with cOmplete™ EDTA-free protease inhibitor cocktail (Roche Molecular Biochemicals, Laval, Quebec, Canada) and PhosSTOP™ phosphatase inhibitor (Roche Molecular Biochemicals). The solution was clarified by centrifugation at 14000 g for 15 min, and the supernatant was stored in aliquots at − 80 °C. For the western blotting assay, protein lysate was size-fractionated by 10% sodium dodecyl sulfate (SDS)-polyacrylamide gel electrophoresis (PAGE) gel, blotted onto a polyvinylidene difluoride (PVDF) membrane (Bio-Rad, Hercules, CA, USA) and blocked with BlockPRO™ 1 Min Protein-Free Blocking Buffer (Energenesis Biomedical, Taipei City, Taiwan) for 3 min. The blots were subsequently probed at 4 °C overnight with antibodies against NF-κB p65 (1 in 1000 dilution in blocking buffer; Abcam). Following incubation with horseradish peroxidase-conjugated secondary anti-rabbit antibody (1 in 10,000 dilution in blocking buffer; Jackson ImmunoResearch Laboratories, Philadelphia, PA, USA), the protein bands were visualized with the Clarity™ Western ECL Blotting Substrates (Bio-Rad) and detected by ChemiDoc™ Imaging Systems (Bio-Rad).

### Histopathological and immunohistochemistry scoring

We used a soft-tissue sarcoma grading system. The sarcomas were scored as 1 to 3 for cell differentiation (1 = mature differentiation; 2 = defined differentiated types; 3 = poor differentiation); mitotic rates (1 = less than or equal to 9 mitoses per 10 400 X fields; 2 = 10–19 mitoses per 10 400 X fields; 3 = more than or equal to 20 mitoses per 10 400 X fields), and necrosis (1 = no necrosis; 2 = necrosis of less than 50% of total area; 3 = necrosis of more than 50% of total area). Final scores of less than 4, between 5 and 6, and more than 7 were designated as grade I, II, and III, respectively [5]. In addition, immunohistochemistry scores were designated as either positive (+) or negative (−) for unequivocal brown nuclear NF-κB staining in at least 5% of tumor cells as well as lymphoid aggregates surrounding the neoplasm [50]. All samples were anonymized and independently scored by two pathologists (C.-S. H. and H.-W. C.). Both pathologists agreed on all results.

### Immunocytochemistry staining (ICC)

Immunocytochemistry staining was used to characterize the phenotypes of the primary cell cultures with their corresponding FFPE section cells, and to confirm if NF-κB inhibitor, DHMEQ, would inhibit nuclear translocation of p65 NF-κB in cell culture or not. The neoplastic cells were treated or not with DHMEQ for 3 h and subsequently fixed with 1:1100% acetone / 100% methanol fixative (Merck, Darmstadt, Germany) for 20 min at − 20 °C. The fixative was aspirated and cells rinsed with PBS 5 times. Various antibodies (NF-κB p65, α-SMA and desmin; 1:200 dilution) applied to the cells were incubated for 1 h at room temperature. The cells were then washed with PBS six times. The primary antibodies were detected by a REAL Envision kit (Dako) using diaminobenzidine as a chromogenic substrate. The cells were counterstained with hematoxylin (Muto Pure Chemicals) for 10 s. Negative controls were created by omitting the primary antibodies. All FISS-07, − 08 and − 10 treated with DHMEQ with subsequent immunocytochemistry staining were performed with triple independent experiments.

## Reagents

Hydroxymethylepoxyquinomicin ((−)-DHMEQ; MedChem Express, Monmouth Junction, NJ, USA), which is an NF-κB inhibitor, was commercially obtained, and was dissolved in dimethylsulfoxide (DMSO; Mallinckrodt Chemical Works, St Louis, MO, USA).

### Inhibition of cell proliferation assay

The effect of the NF-κB inhibitor DHMEQ on neoplastic cell proliferation was evaluated using AlamarBlue Cell Viability Assay Reagent (G-Biosciences, St. Louis, MO, USA) at 24, 48, and 72 h. Cells were seeded on 96-well plates (4.5 × 10^3^ cells/well, CORNING) for 24 h at 37 °C and subsequently replenished with fresh medium with various concentrations of DHMEQ. Control cells were only treated with either culture medium, or culture medium containing DMSO, at a concentration used as DHMEQ diluent. Control cells incubated with medium alone were used to standardize relative viable cell numbers. Three independent experiments for each primary cell line were carried out using three replicates for each drug concentration. The IC50 value was defined as the concentration of DHMEQ, which inhibited cell growth by 50%. Dose-dependent nonlinear regression analysis fitting to a sigmoid dose–response curve was generated by PRISM software (PRISM 6, GraphPad Software, La Jolla, CA, USA).

### Clonogenic assay

The effect of DHMEQ on the clonogenic ability of neoplastic cells was evaluated. Single cell suspensions of 450 cells/well were seeded in six-well plates (CORNING) and incubated for 2 h for cell attachment. The cells were then treated with DHMEQ at concentrations of 20, 10, 5, and 2.5 μg/ml for 72 h. Control cells incubated with culture medium containing DMSO at a concentration used as DHMEQ diluent were used to standardize relative number of colony formation. After the treatment, the cells were refreshed with drug-free culture medium and incubated at 37 °C for approximately 3 weeks until the control dishes had formed sufficiently large clones. Every 3–5 days, the medium was removed and replaced with fresh medium. Finally, the cells were washed with PBS and stained with 0.1% crystal violet solution in 20% ethanol (Sigma Aldrich, Steinheim, Germany) at room temperature for 10 min. Colonies with more than 30 cells were counted. Plating efficiency (PE) and survival fraction (SF) were calculated based on a previous study [51]. Cells treated with each drug concentration were tested in quintuplicate and three times independently.

### Cell migration for wound healing assay

Briefly, cells (4.5 × 10^4^ cells/well) were seeded into 24-well plates (CORNING), supplied with DMEM containing 1% fetal bovine serum and incubated at 37 °C overnight. Wounds were generated by scratching using a pipette tip on confluent cell monolayers. The wounds were measured and photographed at time 0. Cells were then treated with DHMEQ at concentrations of 20, 10, 5, and 2.5 μg/ml, or DMSO alone as the vehicle control, and allowed to migrate toward the denuded areas for 24 h. Cell migration over distances were photographed and compared to a control group. The distances were measured in micrometers using ImageJ (NIH, Bethesda, MD, USA). At least 50 different measurements per treatment were performed to obtain an average index. Data are the averages of triplicate determinations from three independent experiments.

### Apoptosis assessment by terminal deoxynucleotidyl transferase dUTP nick end labeling (TUNEL) assay

Cell death was assessed by the DeadEnd™ Fluorometric TUNEL System (Promega, Madison, WI, USA) according to the manufacturer’s instruction. Approximately 4.5 × 10^4^ neoplastic cells were plated in 24-well plates and incubated at 37 °C overnight. On the next day, the cells were treated with fresh medium containing various concentrations of DHMEQ and DMSO alone and were evaluated by the TUNEL assay 24 h post drug administration. To collect the cells, the supernatant medium was removed and the cells were washed with cold PBS and the trypsinized cells were pooled and centrifuged on the slide at 800 rpm for 8 min by the Shandon CytoSpin 3 (Thermo Shandon, Pittsburgh, PA, USA). The slides were processed for TUNEL staining. Eight areas for each stained section were randomly selected and photographed at × 200 with an Olympus IX83 epifluorescent microscope (Olympus, Center Valley, PA, USA). The mean number of positive cells per treatment was calculated using ImageJ. Data are obtained from three independent experiments, with triplicate determinations in each experiment.

### Statistical analysis

All statistical tests were performed using SPSS version 20 (SPSS, Chicago, IL, USA). Fisher’s exact test was used to examine the association between expression of NF-κB p65 and the histopathological grading and clinical variables. For in vitro studies, *P* < 0.05 indicates significant difference, and analyses were conducted by one-way analysis of variance with Bonferroni’s post hoc correction. Statistical significance included comparisons between treated and untreated groups. Results are expressed as means ± standard deviation.

## Data Availability

The datasets used and/or analyzed during the current study are available from the corresponding author on reasonable request.

## Abbreviations

DHMEQ: Dehydroxymethylepoxyquinomicin
DMSO: Dimethylsulfoxide
FFPE: Formalin-fixed paraffin-embedded
FISS: Feline injection sites sarcomas
HIF-1: Hypoxia-inducible factor 1
IC50: Half maximal inhibitory concentration
ICC: Immunocytochemistry
IHC: Immunohistochemistry
IKK: IκB kinase
IL-1: Interleukin-1
LPS: Lipopolysaccharides
NF-κB: Nuclear factor-kappa B
PBS: Phosphate buffered saline
PE: Plating efficiency
PVDF: Polyvinylidene difluoride
SD: Standard deviation
SDS-PAGE: Sodium dodecyl sulfate polyacrylamide gel electrophoresis
SF: Survival fraction
STAT-3: Signal transducer and activator of transcription 3
TNFα: Tumor necrosis factor α
TUNEL: Terminal deoxynucleotidyl transferase dUTP nick end labeling
VAS: Vaccine-associated sarcomas
α –SMA: α-smooth muscle actin
